# Abnormal Expression of Centromere Protein U Is Associated with Hepatocellular Cancer Progression

**DOI:** 10.1155/2021/4051192

**Published:** 2021-12-11

**Authors:** Yuanlin Yu, Xiaopeng Chen, Weidong Zhang, Jun Liu

**Affiliations:** ^1^Department of Liver Transplantation and Hepatobiliary Surgery, Shandong Provincial Hospital, Cheeloo College of Medicine, Shandong University, Jinan, Shandong 250012, China; ^2^Department of Hepatobiliary Surgery, First Affiliated Hospital of Wannan Medical College, Wuhu, Anhui 241001, China

## Abstract

**Background:**

Hepatocellular carcinoma (HCC) is one of the most common malignancies globally, but its molecular mechanism is unclear. Abnormal expression of centromere protein U (CENPU) is closely related to diverse human cancers. The purpose of this article was to evaluate the function and potential mechanisms of CENPU in HCC development.

**Methods:**

We performed bioinformatics analysis of The Cancer Genome Atlas (TCGA), Gene Expression Omnibus (GEO), Gene Expression Profiling Interactive Analysis (GEPIA), and Kaplan-Meier plotter databases to investigate the clinical significance and prognostic value of CENPU in HCC. Western blotting and immunohistochemical staining were used to measure protein expression, while reverse transcription-quantitative polymerase chain reaction (qRT-PCR) was used to determine mRNA expression. Cell Counting Kit8 (CCK-8) and colony formation assays were conducted to examine cell proliferation. Transwell and wound healing assays were used to assess cell migration and invasion. Gene set enrichment analysis (GSEA) was used to explore the potential signaling pathways of CENPU involved in HCC.

**Results:**

High expression of CENPU in HCC was predicted by public database analysis and indicated a poor prognosis. CENPU expression was significantly higher in HCC tissues and cells than in normal tissues and cell. In vitro, CENPU promoted the proliferation, migration, and invasion of HCC cells. GSEA results indicated that CENPU was linked to the Notch signaling pathway, and our research supported this prediction.

**Conclusion:**

CENPU promotes the malignant biological process of HCC and may be a promising target for HCC treatment.

## 1. Introduction

Hepatocellular carcinoma (HCC) is a common malignancy with high morbidity and mortality rates worldwide [1–3]. Currently, the standard treatments for HCC patients mainly involve liver resection, liver transplantation, radiofrequency ablation, transarterial chemoembolization, and chemotherapy [4–7]. Although considerable effort has been devoted to the study of surgical procedures or chemical therapies, the 5-year survival rates of patients with HCC in China have been reported to be as low as 12% [8]. Therefore, exploring new effective treatment modalities for HCC has crucial clinical significance.

Centromere protein U (CENPU), also called CENP-50/PBIP1, KLIP1, or MLF1IP, is exclusively expressed in centromeres throughout the cell cycle [9–15]. Recently, multiple studies have suggested the aberrant expression of CENPU in various human solid tumors, such as non-small-cell lung cancer (NSCLC), bladder cancer, ovarian cancer, and breast cancer, which highlights the crucial role of CENPU in these tumors [16–20]. Cui et al. have found that the expression of CENPU mRNA was significantly higher in HCC than in normal tissues by using the Oncomine and Gene Expression Profiling Interactive Analysis (GEPIA) databases [21]. Nevertheless, the potential value of CENPU in HCC has not yet been experimentally investigated previously.

Notch signaling is a crucial pathway in tumorigenesis through the regulation of cell proliferation, apoptosis, and differentiation [22]. Villanueva et al. reported that Notch signaling is activated in human HCC samples and promotes formation of liver tumors in mice [23]. However, the relationship between CENPU and Notch signaling pathway in HCC remains unexplored.

In this study, we first explored the expression and clinical significance of CENPU through bioinformatics analysis, which suggested that CENPU is overexpressed in HCC tissues and that high CENPU expression is correlated with poor prognosis among patients with HCC. Subsequently, our data indicated that CENPU is highly expressed in human HCC tissues and cell lines, which was consistent with the results of the public database analysis. We also explored the biological role of CENPU in human HCC cells and demonstrated that knockdown or overexpression of CENPU has been shown to inhibit or enhance HCC cell proliferation, migration, and invasion, respectively. Moreover, our study indicated that CENPU may be involved in the regulation of Notch signaling pathway in HCC. Consequently, our study uncovered CENPU as a potential prognostic marker and a potential therapeutic target for HCC.

## 2. Materials and Methods

### 2.1. Biological Information Analysis

The expression of CENPU in common digestive cancers and HCC was predicted using an online website (http://www.aclbi.com/) based on The Cancer Genome Atlas (TCGA) and Genotype-Tissue Expression (GTEx) databases. GSE84402, GSE14520, and GSE46408 datasets were downloaded from the Gene Expression Omnibus (GEO) database. GSE84402 has information on 14 cancer tissues and 14 adjacent normal tissues. GSE14520 has information on 23 cancer tissues and 20 adjacent normal tissues. GSE46408 has information on 6 cancer tissues and 6 adjacent normal tissues. A total of 374 HCC tissue samples and 50 normal liver normal tissue samples with corresponding clinicopathological information and RNA-Seq expression data were downloaded from TCGA database (https://portal.gdc.cancer.gov/). We used GEPIA (http://gepia.cancer-pku.cn/) to analyze overall survival and disease-free survival rate of patients with HCC. We used Kaplan-Meier Plotter (http://kmplot.com/) to analyze overall survival, disease-free survival, progression-free and relapse-free survival. Data processing and analysis were performed using the R language (version 4.0.3) and Perl programming language (version 5.32.1). Gene set enrichment analysis (GSEA) was performed using GSEA software (version 4.1.0).

### 2.2. Cell Lines and Clinical Specimens

Five human HCC cell lines Bel7402, SMCC7721, HepG2, HCCLM3, and Huh7 and the human normal liver cell line LO2 were purchased from the Cell Bank of Shanghai Academy of Chinese Science. The cells were cultured in RPMI 1640 medium (Thermo Fisher Scientific, USA) and DMEM (HyClone, USA) with 10% FBS (Thermo Fisher Scientific, USA). All cells were grown at 37°C and 5% carbon dioxide. Ten fresh HCC tissues and paracancerous tissues were collected from patients at the First Affiliated Hospital of Wannan Medical College. Each patient signed an informed consent form, and the study was approved by the Medical Ethics Committee of the First Affiliated Hospital of Wannan Medical College. The study was performed in accordance with the Declaration of Helsinki.

### 2.3. Cell Transfection

The CENPU RNAi plasmid and corresponding empty vector, as well as CENPU-shRNA and a nonspecific control pool (negative control), were purchased from GeneChem Biotechnology (Shanghai, China). Human HCC cell lines in the exponential growth period were inoculated in six-well plates. For overexpression and knockdown assays, 2.5 *μ*g plasmids were transfected with Lipo8000™ Transfection Reagent (Beyotime, China) into the cells according to the reagent instructions. Transfection efficiency was confirmed by fluorescence microscopy and western blotting. The target sequence for CENPU-shRNA1 was 5′-AAGCTCAAGAACCAAACGTAA-3′. The target sequence for CENPU-shRNA2 was 5′-ACCCACCTAGAGCATCAACAA-3′. The target sequence for CENPU-shNC was 5′-TTCTCCGAACGTGTCACGT-3′. For convenience, we marked them as sh1 and sh2 and NC, respectively.

### 2.4. Western Blotting

Tissues or cells were lysed with RIPA containing protease inhibitors (Beyotime, China) on ice for 30 min. Samples were separated on SDS-PAGE gels and blotted onto PVDF membranes. After being blocked with blocking buffer (Biosharp, China) for 1 hour, the PVDF membranes were incubated with the following diluted primary antibodies at 4°C overnight: anti-CENPU (Affinity, China, 1 : 1000), anti-Notch1 (Abcam, USA, 1 : 1000), anti-Hes1 (Abcam, USA, 1 : 1000), anti-Hey1 (Abcam, China, 1 : 1000), and anti-*β*-actin (Affinity, China, 1 : 2000). Then, the PVDF membranes were incubated with specific horseradish peroxidase- (HRP-) conjugated IgG secondary antibodies (Affinity, 1 : 2000). The protein bands on the PVDF membrane were visualized by using a Tanon 5200 Imaging System.

### 2.5. RNA Extraction and qRT-PCR

Total RNA was isolated from cells or tissue by Trizol agent (Tiangen, China). Ten nanograms of total RNA was reverse transcribed into cDNA using the Fast King One Step RT-PCR Master Mix kit (Tiangen, China). qPCR reactions were performed with SYBR Green Real-time PCR Master Mix (Tiangen, China) using the following PCR primers: CENPU forward, ACCCACCTAGAGCATCAACAA; CENPU reverse, ACTTCAATCATACGCTGCCTTT; GAPDH forward, TGTGGGCATCAATGGATTTGG; and GAPDH reverse, ACACCATGTATTCCGGGTCAAT. Then, a QuantStudio™ 3 Real-Time PCR System (Thermo Fisher Scientific, USA) was used to complete the PCR amplification. CENPU mRNA was calculated using the 2-*ΔΔ*CT method.

### 2.6. Immunohistochemistry (IHC) Staining

HCC tissues and adjacent tissues were fixed, embedded in paraffin, and cut into 4 *μ*m sections. Then, the samples were heated at 100°C for 15 min incitric acid buffer for antigen retrieval. CENPU antibody (Affinity, 1 : 200) was added as the primary antibody and incubated at 4°C overnight. The following day, the sections were incubated with fluorescence-labeled secondary antibodies (Affinity, China) and finally stained with the diaminobenzidine (DAB) method (Beyotime, China). The degree of IHC staining was scored by two independent pathologists.

### 2.7. CCK-8 Assay

A total of 2 × 10^3^ cells per well were cultured and seeded into 96-well plates for 0, 24, 48, and 72 hours. Ten microliters of CCK-8 reagent (BestBio, China) was added to each well, and the cells were cultured for 2 hours. The absorbance at 450 nm was recorded using an automated microplate reader (BioTek Instruments, USA).

### 2.8. Colony Formation Assay

A total of 1 × 10^3^ cells were added to a 6-well plate and incubated for two weeks. Then, the colonies on the plates were fixed using 4% paraformaldehyde and stained with 0.1% crystal violet solution, and the colonies were imaged and quantified.

### 2.9. Wound Healing Assay

Cells were seeded into a 6-well plate and allowed to reach approximately 90% confluence. A linear wound was scratched across the cell monolayer using a 200 *μ*L tip. The cells were observed and photographed at 0 hours and 48 hours using an inverted microscope.

### 2.10. Transwell Assay

Transwell chambers (Corning, USA) were used to determine cell invasion and migration ability. A total of 5 × 10^4^ cells/well were plated into the upper chamber, and the lower chamber was maintained with 10% FBS medium. After 24 hours of culture, invading and migrating cells were stained with 0.1% crystal violet and imaged under an inverted microscope.

### 2.11. Statistical Analysis

All data were analyzed using GraphPad Prism and expressed as the mean ± SD. Comparisons between two groups were assessed by Student's *t*-test or *χ*^2^ test. Univariate and multivariate Cox regression analyses were used to analyze the prognosis. Difference with *P* values less than 0.05 were reported as statistically significant.

## 3. Results

### 3.1. CENPU Expression in Public Datasets

**Figure 1:**
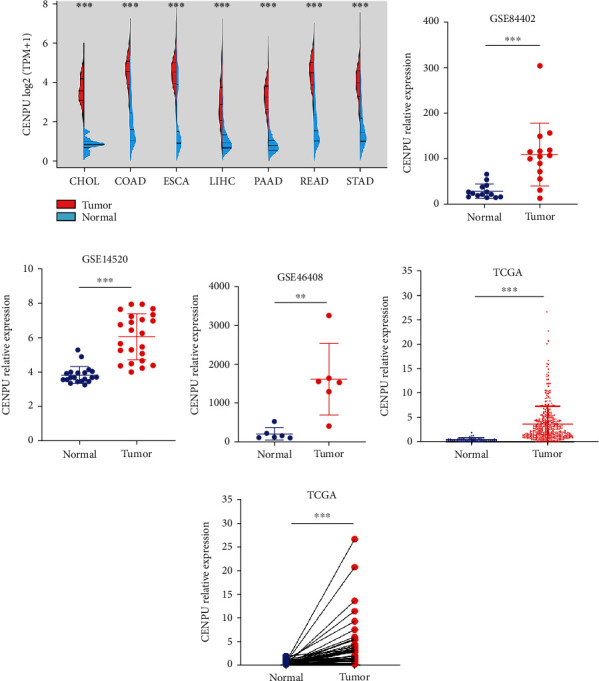
*CENPU expression in public databases*. (a) TCGA and GTEx dataset analysis showed that CENPU was upregulated in digestive system cancers. (b–d) CENPU was elevated in HCC tissues compared with normal liver tissues from the GSE84402, GSE14520, and GSE46408 based on GEO datasets. (e) CENPU expression was significantly higher in HCC tissues than normal liver tissues according to TCGA HCC database (374 tumor and 50 normal tissues). (f) CENPU expression was significantly higher in 50 cases HCC tissues than in the matched normal liver tissues. ^∗∗^*P* < 0.01 and ^∗∗∗^*P* < 0.001.

We first analyzed the expression of CENPU in common digestive cancers in TCGA and GTEx datasets. The results showed that CENPU expression was upregulated in esophageal cancer, stomach cancer, pancreatic cancer, HCC, colon cancer, and rectal cancer tissues compared with normal tissues (Figure 1(a)). CENPU expression was elevated in HCC tissues relative to normal liver tissues in GEO datasets (Figures 1(b)–1(d)). By analyzing the mRNA sequencing datasets from TCGA HCC cohort, we identified that CENPU was elevated in human HCC tissues versus normal liver tissues (Figures 1(e) and 1(f)). As shown in Table 1, overexpression of CENPU was significantly correlated with histologic grade (*P* = 0.002), tumor-node-metastasis (TMN) stage (*P* = 0.001), and tumor depth (*P* < 0.001), while overexpression of CENPU was not connected with age, sex, M, and N classification (Table 1).

### 3.2. The Prognostic Value of CENPU according to the Public Database Cohort

**Figure 2:**
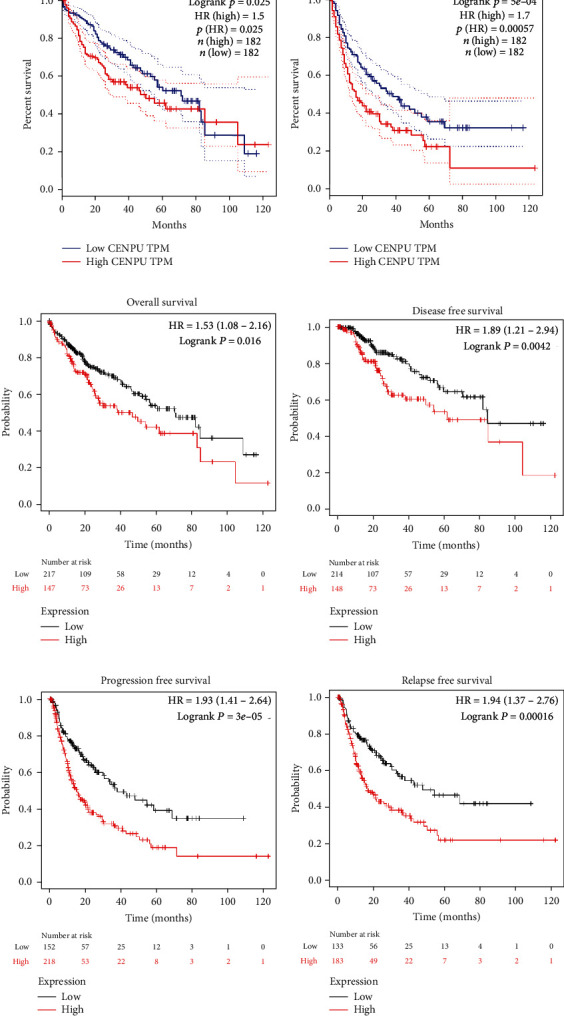
Prognostic analysis of CENPU expression according to public database cohort. (a, b) Kaplan-Meier curves of CENPU for overall survival and disease-free survival data were computed by GEPIA. (c) Overall survival, (d) disease-free survival, (e) progression-free survival, and (f) relapse-free survival analysis of HCC patients with the Kaplan-Meier plotter online tool.

GEPIA and Kaplan-Meier plotter online tools were used to evaluate the prognostic value of CENPU. We observed that high CENPU expression was inversely correlated with overall survival (*P* = 0.025) and disease-free survival (*P* < 0.001) in HCC patients according to the GEPIA website (Figures 2(a) and 2(b)). Additionally, Kaplan-Meier plotter analysis revealed that high CENPU expression had significant value for predicting unfavorable overall survival (*P* = 0.016, Figure 2(c)), disease-free survival (*P* = 0.004, Figure 2(d)), progression-free survival (*P* < 0.001, Figure 2(e)), and relapse-free survival (*P* < 0.001, Figure 2(f)). Subsequently, univariate and multivariate Cox regression analyses were conducted to identify independent predictors for overall survival in TCGA HCC database, and revealed that TMN stage (*P* < 0.001), tumor depth (*P* < 0.001), distant metastasis (*P* = 0.023), and CENPU expression (*P* = 0.005) were prognostic factors for overall survival in TCGA HCC cohort. Furthermore, multivariate analysis data indicated that CENPU expression (*P* = 0.045) was an independent poor prognostic factor for HCC patients (Table 2).

### 3.3. CENPU Expression Status in HCC Tissues and Cell Lines

**Figure 3:**
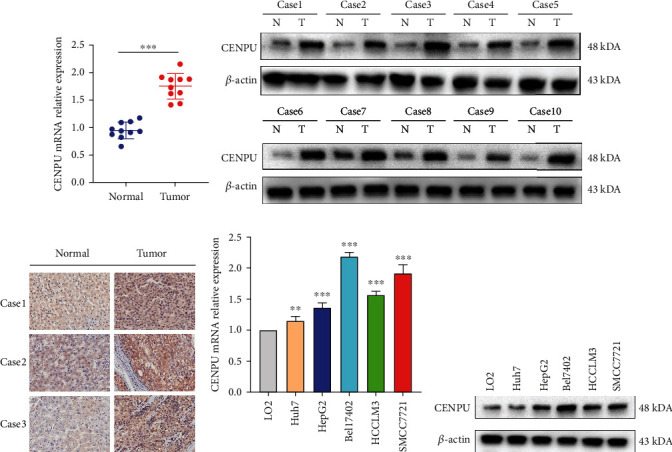
CENPU expression in HCC tissues and cell lines. (a, b) CENPU mRNA and protein expression levels were quantified in HCC tissues and adjacent normal liver tissues by qRT-PCR and western blot. (c) CENPU expression in HCC tissues and adjacent normal liver tissues was detected by IHC (magnification, ×200). (d, e) qRT-PCR and western blot analysis were conducted to measure CENPU mRNA and protein expression in LO2 normal liver cells and HCC cell lines. ^∗∗^*P* < 0.01 and ^∗∗∗^*P* < 0.001.

As mentioned previously, CENPU expression was elevated in HCC tissues compared with normal liver tissues in public datasets. We initially investigated the expression of CENPU in our fresh HCC tissues and adjacent normal liver tissues, and the results suggested that CENPU expression was elevated in HCC tissues relative to adjacent normal liver tissues (Figures 3(a)–3(c)). Consistently, we detected the expression of CENPU in LO2 normal liver cells and HCC cell lines including HepG2, Huh7, SMCC7721, Bel7402, and HCCLM3. The results showed that the expression of CENPU was elevated in HCC cell lines compared with the LO2 cell line, especially in Bel7402 and SMCC7721 cells (Figures 3(d) and 3(e)). Hence, these two cell lines were used for subsequent experiments.

### 3.4. CENPU Knockdown Suppresses HCC Cell Proliferation, Migration, and Invasion

**Figure 4:**
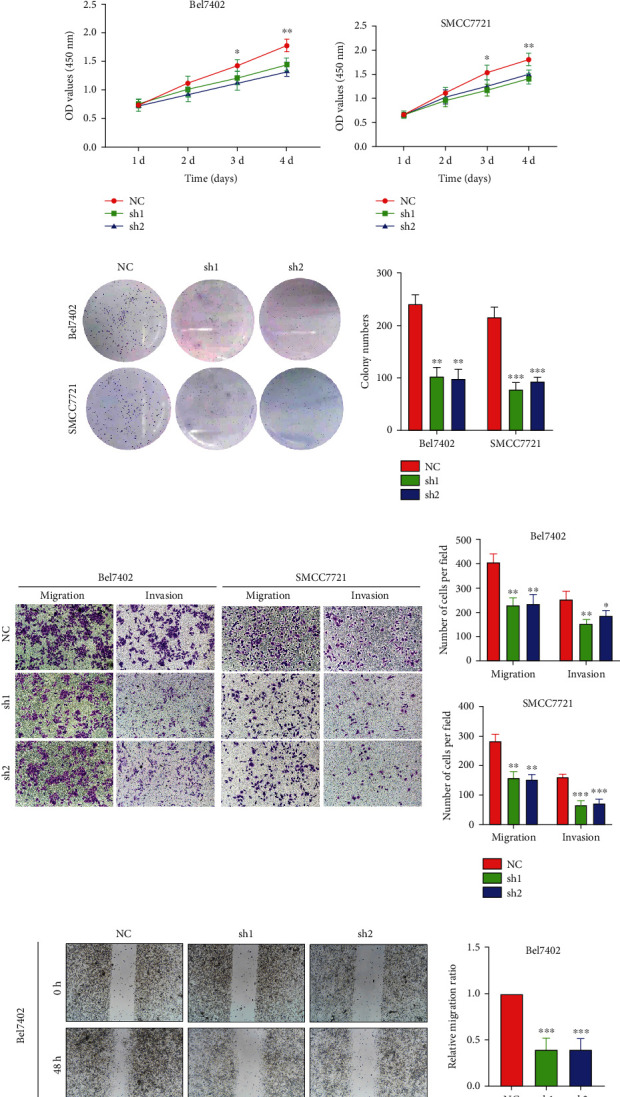
CENPU knockdown suppresses HCC cell proliferation, migration, and invasion. (a, b) The CENPU knockdown of efficiency in Bel7402 and SMCC7721 cell lines was assessed by fluorescence microscopy (magnification, ×100) and western blotting. (c–e) CCK-8 and colony formation assays showed that CENPU knockdown suppressed the proliferation of Bel7402 and SMCC7721 cells. (f, g) Transwell (magnification, ×100) and scratch assays (magnification, ×40) showed that knockdown of CENPU inhibited the migration and invasion of Bel7402 and SMCC7721 cells. ^∗^*P* < 0.05,  ^∗∗^*P* < 0.01, and^∗∗∗^*P* < 0.001.

As presented above, CENPU expression was significantly associated with the prognosis of HCC patients. Accordingly, we hypothesized that downregulation of CENPU would suppress the malignant behaviors of HCC cells. To explore the potential biological function of CENPU, we selected Bel7402 and SMCC7721 cells as CENPU knockdown models. Knockdown effectiveness was evaluated by western blotting and fluorescence microscopy (Figures 4(a) and 4(b)). CCK-8 assay (Figures 4(c) and 4(d)) and colony formation assay (Figure 4(e)) indicated that knockdown of CENPU significantly inhibited the proliferation of the Bel7402 and SMCC7721 cells. Subsequently, Transwell assays (Figure 4(f)) and wound healing assays (Figure 4(g)) proved that CENPU downregulation suppressed the migration and invasive capability of Bel7402 and SMCC7721 cells.

### 3.5. CENPU Overexpression Promotes HCC Cell Proliferation, Migration, and Invasion

**Figure 5:**
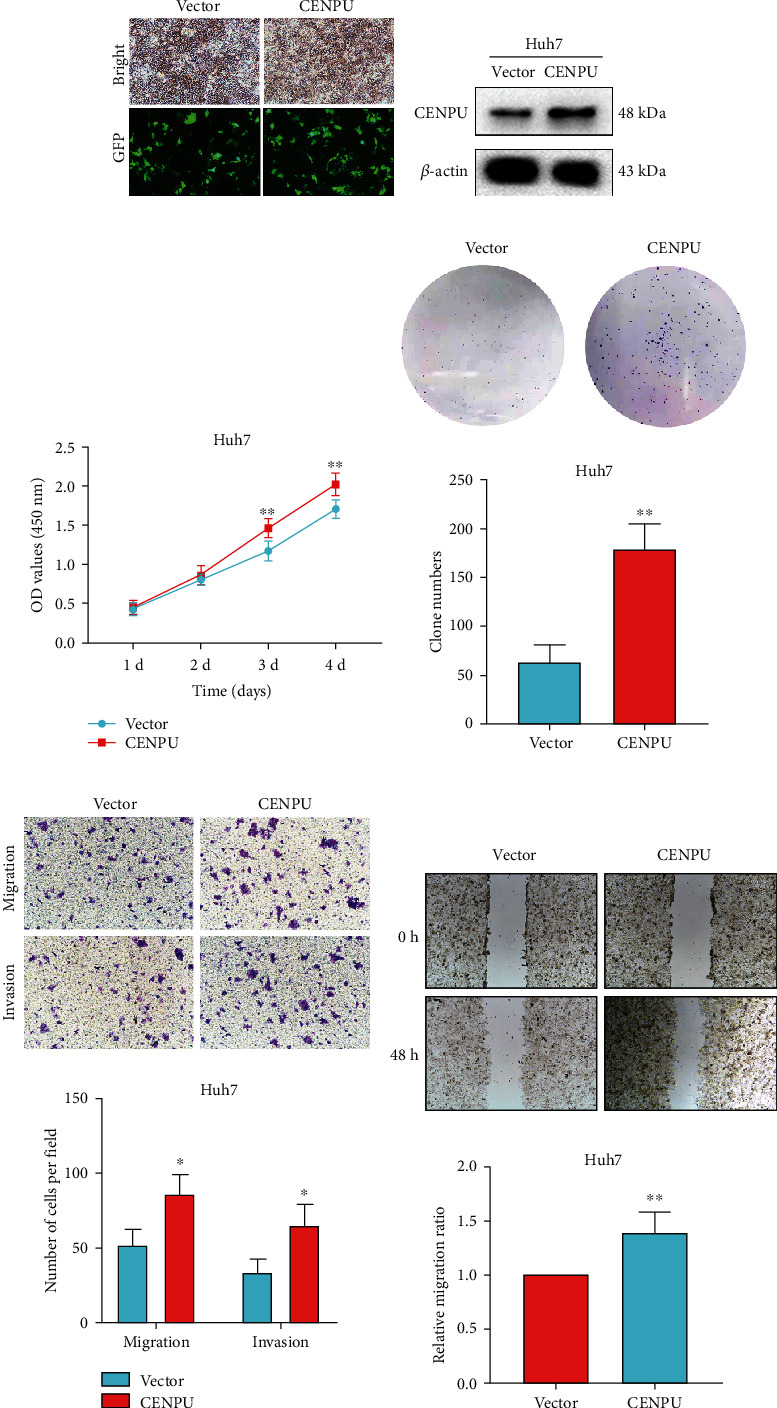
Overexpression of CENPU promotes HCC cell proliferation, migration, and invasion. (a, b) CENPU overexpression of efficiency in Huh7 cell line was assessed by fluorescence microscopy (magnification, ×100) and western blotting. (c, d) CCK-8 and colony formation assays showed that CENPU overexpression promoted Huh7 cell proliferation. (e, f) Transwell (magnification, ×100) and scratch assays (magnification, ×40) showed that overexpression of CENPU enhanced Huh7 cell migration and invasion. ^∗^*P* < 0.05 and^∗∗^*P* < 0.01.

The CENPU overexpression plasmid was transfected intoHuh7 cells, and fluorescence microscopy and western blotting were used to confirm the overexpression efficiency (Figures 5(a) and 5(b)). The CCK-8 assay indicated that overexpression of CENPU increased the proliferation ability of Huh7 cells (Figure 5(c)). Colony formation assays indicated that CENPU overexpression promoted Huh7 cell proliferation (Figure 5(d)). Transwell assays revealed that CENPU overexpression promoted Huh7 cell migration and invasion (Figure 5(e)). Then, a wound healing assay showed that CENPU overexpression markedly promoted Huh7 cell migration (Figure 5(f)).

### 3.6. CENPU Modulates the Notch Signaling Pathway

**Figure 6:**
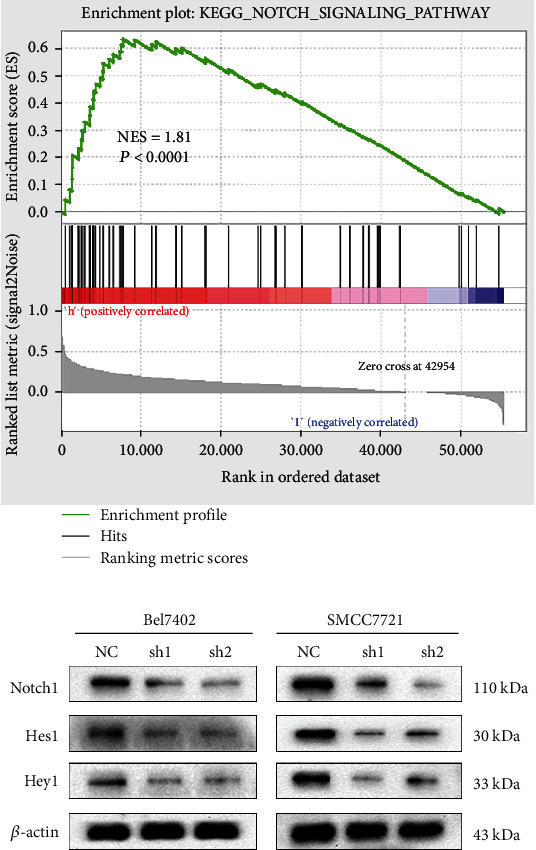
CENPU modulates the Notch signaling pathway. (a) GSEA indicated that CENPU was significantly correlated with the Notch signaling pathway. (b) Western blotting analysis of the expression of the Notch signaling-related proteins Notch1, Hes1, and Hey1 in Bel7402 and SMCC7721 cell lines.

To evaluate the possible underlying molecular mechanism of CENPU in the progression of HCC, GSEA was conducted to identify the signaling pathway of CENPU in HCC. GSEA results showed that CENPU overexpression was positively associated with Notch signaling pathway (Figure 6(a)). Therefore, we examined the major proteins such as Notch1, Hes1, and Hey1 in the Notch pathway by western blotting. Inhibition of CENPU expression in Bel7402 and SMCC7721 cells decreased the expression of these proteins in the Notch signaling pathway (Figure 6(b)). These results revealed that high expression of CENPU in HCC may activate the Notch signaling pathway.

## 4. Discussion

Hanissian et al. [9] first reported that CENPU was correlated with myeloid leukemia factor 1 (MLF1) and located in both the nuclei and cytoplasm of cells. CENPU localizes to human chromosome 4q35.1 and encodes a 46 kDa protein [10]. More recently, previous studies suggested that CENPU was expressed in various tissues such as a fetal liver, bone marrow, and thymus and testis [11–15]. Accumulating evidence has indicated that CENPU is upregulated in several human cancers and may play a key role in cancer progression [16–20]. Studies have revealed that CENPU expression is highly expressed in NSCLC tissues and that CENPU knockdown represses tumor proliferation and metastasis in NSCLC cells by regulating the Wnt/*β*-catenin signaling pathway [16]. Additionally, Wang et al. [19] reported that CENPU is upregulated in NSCLC tissues and facilitates lung cancer cell proliferation by targeting the transcription factor FOXM1. CENPU is elevated in human bladder cancer tissues compared with surrounding tissues, and high CENPU expression is obviously associated with tumor size, TNM stage, and poor prognosis [18]. Recent researchers revealed that CENPU highly expressed in ovarian cancer samples and that overexpression of CENPU augments ovarian cancer aggressiveness by regulating high-mobility group box 2 (HMGB2) [17]. Pan et al. [20] demonstrated that CENPU promotes tumor angiogenesis through the cyclooxygenase-2- (COX-2) mediated signaling pathway in triple-negative breast cancer. Nevertheless, the functional role of CENPU in HCC remains unclear.

In this study, we first revealed that CENPU is upregulated in common digestive cancer tissues compared to normal tissue through bioinformatics analysis. Therefore, the oncogenic value of CENPU in HCC was explored for a further study. Bioinformatics analysis confirmed that high CENPU expression is related to a poor prognosis in HCC patients. Additionally, univariate and multivariate analyses showed that TMN stage, tumor depth, distant metastasis, and CENPU expression can be used as independent risk factors for HCC. These results indicated a possible function of CENPU in HCC pathogenesis.

To the best of our knowledge, this is one of the first studies to determine CENPU expression in HCC cells and explore its clinical value among patients with HCC. Consistent with public databases, our data indicated that CENPU is highly expressed in HCC tissue, whereas in adjacent noncancerous tissues CENPU expression is low. Moreover, HCC cell lines showed higher expression of CENPU than a normal liver cell line. In the current study, we conducted in vitro assays to investigate the effect of CENPU on tumor development. Furthermore, our results revealed that downregulation of CENPU restrained HCC cell proliferation, migration, and invasion, while upregulation of CENPU facilitated HCC cell proliferation, migration, and invasion. Our findings indicated that CENPU has a tumorigenic role in the development of HCC.

To further study the molecular mechanism of CENPU in HCC, we performed GSEA to investigate potential related signaling pathways. The GSEA results revealed that CENPU expression was associated with the Notch signaling pathway. Some studies have found that the Notch signaling pathway regulates cell differentiation in addition to cell proliferation and metastasis in a variety of cancers [24–27]. We demonstrated that the expression of Notch signaling pathway proteins including Notch1, Hes1, and Hey1 was significantly decreased after CENPU knockdown in HCC cells. Together, these findings indicated that CENPU might be an oncogene that promotes HCC progression through the Notch signaling pathway. Hence, further research on this topic is recommended in the future.

## 5. Conclusions

In summary, our findings indicate that CENPU is upregulated in HCC and is an unfavorable predictor of prognosis in HCC patients. Furthermore, CENPU may promote HCC cell proliferation, migration, and invasion through the Notch signaling pathway. Therefore, targeting CENPU might represent a novel therapeutic strategy for HCC patients.

## Figures and Tables

**Table 1:** Clinicopathologic features of CENPU expression in TCGA HCC cohort.

Characteristics	CENPU expression		Cases	*P*
	High expression	Low expression		
Age (years)	117	117		0.236
≤60	70	61	131	
>60	47	56	103	
Gender				0.092
Female	43	31	74	
Male	74	86	160	
Histologic grade				0.002^∗^
G1+G2	54	78	132	
G3+G4	63	39	102	
TNM stage				0.001^∗^
I	52	61	113	
II	25	24	49	
III+IV	40	29	67	
T classification				
T1	52	63	115	<0.001^∗^
T2	26	25	51	
T3+T4	39	29	68	
N classification				0.313
N0	116	114	230	
N1	1	3	4	
M classification				0.313
M0	116	114	230	
M1	1	3	4	

∗ indicates statistical significance.

**Table 2:** Univariate and multivariate analyses of CENPU in TCGA HCC cohort.

Variables	Univariate analysis			Multivariate analysis		
	HR	95% CI	*P*	HR	95% CI	*P*
Age	1.0050	0.9869-1.0235	0.591	1.0092	0.9897-1.0291	0.358
Gender	1.2818	0.8005-2.0526	0.301	0.9062	0.5370-1.5283	0.712
Histologic grade	1.0172	0.7459-1.3871	0.914	1.1063	0.7946-1.5402	0.550
TNM stage	1.8647	1.4558-2.3884	<0.001^∗^	1.2306	0.4535-3.3388	0.684
T classification	1.8044	1.4341-2.2702	<0.001^∗^	1.4276	0.5712-3.5682	0.446
N classification	2.0218	0.4939-8.2761	0.3276	2.0177	0.3435-11.8530	0.437
M classification	3.8498	1.2068-12.281	0.0227^∗^	1.6325	0.4226-6.3068	0.477
CENPU expression	1.4283	1.1118-1.8349	0.0052^∗^	1.3133	1.0056-1.7152	0.045^∗^

∗ indicates statistical significance.

## Data Availability

The datasets used and/or analyzed during the present study are available from the corresponding author on reasonable request.

## Abbreviations

HCC: Hepatocellular carcinoma
CENPU: Centromere protein U
TCGA: The Cancer Genome Atlas
GEO: Gene Expression Omnibus
GTEx: The Genotype-Tissue Expression
CHOL: Cholangiocarcinoma
COAD: Colon Adenocarcinoma
ESCA: Esophageal Carcinoma
LIHC: Liver Hepatocellular Carcinoma
STAD: Stomach Adenocarcinoma
PAAD: Pancreatic Carcinoma
READ: Rectal Adenocarcinoma
GEPIA: Gene Expression Profiling Interactive Analysis
qRT-PCR: Reverse Transcription-quantitative Polymerase Chain Reaction
IHC: Immunohistochemistry
GSEA: Gene Set Enrichment Analysis.
